# High-throughput sequencing for the detection of the bacterial and fungal diversity in Mongolian naturally fermented cow’s milk in Russia

**DOI:** 10.1186/s12866-015-0385-9

**Published:** 2015-02-22

**Authors:** Wenjun Liu, Yi Zheng, Lai-Yu Kwok, Zhihong Sun, Jiachao Zhang, Zhuang Guo, Qiangchuan Hou, Bilige Menhe, Heping Zhang

**Affiliations:** Key Laboratory of Dairy Biotechnology and Engineering, Inner Mongolia Agricultural University, Hohhot, 010018 P. R. China; Synergetic Innovation Center of Food Safety and Nutrition, Northeast Agricultural University, Harbin, 150030 P. R. China

**Keywords:** Pyrosequencing, Naturally fermented cow’s milk, Russia, Kalmykia, Chita, Mongolia

## Abstract

**Background:**

Traditional fermented dairy products are major components of the typical Mongolian diet since ancient times. However, almost all the previous studies on the microbial composition of traditional Mongolian fermented dairy products analyzed food samples from the Chinese Mongolian region and Mongolia but not the Russian Mongolian region. In this study, the bacterial and fungal community diversity of nineteen naturally fermented cow’s milk (NFCM) samples from local Mongolian families residing in Kalmykia and Chita of Russia was investigated with pyrosequencing.

**Results:**

Firmicutes and Ascomycota were the predominant phyla respectively for bacteria and fungi. The abundance of the bacterial phylum Acidobacteria was considerably different between the samples from the two regions. At genus level, *Lactobacillus* and *Pichia* were the predominating bacterial and fungal genera, respectively, while six bacterial genera significantly differed between the Kalmykia (enrichment of *Aeromonas*, *Bacillus*, *Clostridium*, *Streptococcus*, *Vogesella*) and Chita (enrichment of *Lactococcus*) samples. The results of principal coordinate analysis (PCoA) based on the bacterial or fungal composition of the Kalmykia and Chita samples revealed a different microbiota structure between the samples collected in these two locations. The redundancy analysis (RDA) identified 60 bacterial and 21 fungal OTUs as the key variables responsible for such microbiota structural difference.

**Conclusions:**

Our results suggest that structural differences existed in the microbiota of NFCM between Kalmykia and Chita. The difference in geographic environment may be an important factor influencing the microbial diversity of NFCM made by the Mongolians in Russia.

**Electronic supplementary material:**

The online version of this article (doi:10.1186/s12866-015-0385-9) contains supplementary material, which is available to authorized users.

## Background

Many traditional fermented dairy products, such as airag and tarag [1], are regarded as nutraceutics and have been the major components in a typical diet for the Mongol ethnic residing in Mongolia, Kazakhstan, Kyrgyzstan, and some Central Asian regions of Russia since ancient times. These traditional fermented foods remain popular until now. In this study, naturally fermented cow’s milk (NFCM) samples were collected from Mongol-ethnic families of Kalmyk in the Republic of Kalmykia and Buryats in Chita that retain the Mongolian traditional life style and dietary habit. NFCM in these families is a common home-made dairy product, which is a white to yellowish-white liquid fermented from milk with or without specific cultures. NFCM are traditionally made by adding old fermented milk containing the natural starter cultures, thus these products are free from commercial starter cultures and own unique and stable microbial communities. Apart from their role as the NFCM-associated microbial community as a whole, some specific strains isolated from NFCM may have added value as producers of antimicrobial compounds [2] or as probiotics [3]. Although the nutritional value of NFCM is well-known, their microbiota composition has not been fully described. Therefore, it is of interest to further characterize the microbial diversity of NFCM.

The microbial biodiversity of fermented dairy products has been assessed by culture-dependent methods to identify the indigenous bacterial composition. A wide variety of Firmicutes genera, such as *Lactobacillus*, *Lactococcus*, *Streptococcus*, *Leuconoctoc*, which are known to influence the flavor and maturation of fermented food [4,5], have been identified. Some of them have also been isolated from different naturally fermented dairy products [6], including cheese [7], tarag [8], kurut [9], and kefir [10]. Relatively low levels of *Bifidobacteriaceae*, *Moraxellaceae* (mostly *Enhydrobacter*), *Corynebacterium*, *Halomonas*, *Pediococcus*, *Micrococcus* and *Staphylococcus* have been reported as compared with the total bacterial population in fermented dairy products [11].

In order to assess the microbial diversity of fermented dairy products, various culture-independent molecular biology-based techniques have been widely applied, such as denaturing gradient gel electrophoresis (DGGE) [11,12], temporal temperature gel electrophoresis (TTGE) [12], quantitative real-time PCR (qPCR) [13] and Sanger sequencing of 16S rRNA gene clone library [9]. These culture-independent techniques are valuable tools to monitor the microbial structure and dynamics in naturally fermented dairy products. They are not only able to reveal the dominant members present in the samples, but also uncover the rarer bacterial species [14].

Pyrosequencing is an automated high-throughput sequencing technique introduced by Margulies et al [15]. This method is based on the synthesis of single-stranded deoxyribonucleic acid and the detection of the light generated by pyrophosphate released during nucleotide incorporation. This technique is able to analyze the microbial structure, gene content, and hence reveal the microbial-based metabolic potential in an ecosystem through the rapid and accurate sequencing of nucleotide sequences. Pyrosequencing has successfully been used to detect the diversity and dynamics of the bacterial populations in various fermented foods, such as cheese [16,17], kefir [6,18], fermented seafood [19] and fermented soybean [20].

In this study, the microbial populations in NFCM were investigated. Nineteen NFCM samples from The Russian Republics of Kalmykia and Chita were evaluated by ribosomal gene targeted 454-pyrosequencing. However, almost all the previous papers focused on the microbial composition of the Mongolian traditional fermented dairy products analyzed samples prepared by the nomads of the Mongolian region of China and Mongolia [1,21,22] but not those made in the Mongolian region of Russia. Results of this study do not only provide accurate and detailed information on the microbiota diversity of NFCM, but also fill the gap of the study of the traditional Mongolian fermented dairy products.

## Results

### Bacterial and fungal sequence abundance and diversity

A total of 268,549 of bacterial V1-V3 16S rRNA raw sequence reads were generated from the nineteen NFCM samples, with an average of 26,845 sequences read for each sample. Meanwhile, 113,513 of 18S rRNA raw sequence reads were generated for the fungal community. Through PyNAST alignment and 100% sequence identity clustering, the unique representative sequence reads were delimited for further analysis, which corresponded to 15,181 bacterial and 4,490 fungal sequences. The number of unique and classifiable representative OTU sequences for bacteria and fungi were, respectively, 573 (average = 147 OTUs per sample, range = 28-211, SD = 49.36) and 138 (average = 31.7 OTUs per sample, range = 14-56, SD = 13.9) (with high threshold identity at 97% sequence similarity level). Based on homologous sequence alignment method and clustering with information extracted from the RDP and BLAST databases, the lowest level of taxonomy of the identified OTUs was determined. 1.9% of bacterial and 39% of fungal sequences could not be assigned to the genus level.

The Shannon diversity curves but not rarefaction curves for all samples reached the saturation phase (Additional file 1: Figure S1), suggesting that although additional new phylotypes would possibly be identified by increasing the sequencing depth, the majority of bacterial and fungal phylotypes for the samples had already been captured in the current analysis.

The Shannon index, Simpson diversity index, Chao1 and observed species of each sample were used to evaluate the species richness and diversity (Table 1). Mann-Whitney test was applied to assess the richness and diversity of bacteria and fungi between Kalmykia and Chita samples. Since significant differences (p < 0.05) were observed in all indicators of the bacterial community in the two sampling sites, it can be concluded that a large distinction of species richness and diversity of the bacterial community existed between the two groups of samples. On the contrary, there were no statistically significant difference for any of the fungal indicators between samples from the two sampling sites (p > 0.05), indicating that a high similarity of fungal diversity was shared between the two sample groups.

**Table 1:** **Sample information, sequence abundance and microbial diversity in the naturally fermented cow’s milks**

**Sample**	**Sampling location**	**Number of reads**		**Number of OTU**		**Shannon index**		**Simpson index**		**Chao1 index**		**Observed species**	
		**Bacteria**	**Fungi**	**Bacteria**	**Fungi**	**Bacteria**	**Fungi**	**Bacteria**	**Fungi**	**Bacteria**	**Fungi**	**Bacteria**	**Fungi**
E12	Kalmykia	14533	8631	68	21	0.8	1.0	0.2	0.3	93.1	23.8	68.0	21.0
F10	Kalmykia	12367	10087	211	18	3.9	0.5	0.9	0.1	329.0	20.9	210.6	18.0
F2	Kalmykia	15173	8265	168	50	2.5	2.3	0.6	0.7	228.8	98.3	167.8	49.8
F3	Kalmykia	12249	10296	126	31	2.4	0.7	0.6	0.2	193.0	38.4	125.8	30.9
F4	Kalmykia	13465	5854	55	53	1.8	2.5	0.5	0.7	72.2	69.5	54.9	52.9
F6	Kalmykia	18820	8596	29	52	0.5	2.8	0.1	0.7	68.0	61.2	29.0	51.9
F9	Kalmykia	14361	3639	104	56	2.5	2.9	0.7	0.8	141.0	76.1	103.9	55.8
F11	Kalmykia	12640	N.F.	131	N.F.	2.7	N.F.	0.7	N.F.	181.9	N.F.	130.9	N.F.
Ra17	Kalmykia	12215	5545	51	21	1.8	0.4	0.5	0.1	66.0	25.2	51.0	21.0
Mean (Kalmykia samples)		13980.3 ± 2123.5	6768.1 ± 3362.4	104.8 ± 60.1	33.6 ± 19.9	2.1 ± 1.0*	1.5 ± 1.2	0.5 ± 0.3*	0.4 ± 0.3	152.6 ± 89.6*	45.9 ± 31.9	104.7 ± 60.0*	33.5 ± 19.9
D8	Chita	13743	5956	67	17	0.7	0.4	0.2	0.1	84.4	19.2	66.9	17.0
D9	Chita	13379	6426	87	19	1.6	0.7	0.5	0.2	112.1	54.5	86.9	19.0
D12	Chita	13449	N.F.	38	N.F.	0.2	N.F.	0.0	N.F.	50.9	N.F.	38.0	N.F.
E1	Chita	14114	3886	44	14	0.3	0.7	0.1	0.2	96.5	28.0	44.0	13.9
E2	Chita	12510	6020	28	25	0.2	0.8	0.0	0.2	63.5	32.4	28.0	25.0
E3	Chita	9929	8736	57	33	1.8	1.4	0.5	0.5	80.9	84.8	57.0	33.0
E4	Chita	17494	9768	64	36	1.7	1.5	0.5	0.5	77.1	40.8	63.9	36.0
E5	Chita	17059	1240	54	37	2.1	3.4	0.7	0.9	84.2	48.0	53.9	36.8
ELS10	Chita	16157	9999	56	34	1.2	2.0	0.4	0.6	68.2	72.4	56.0	33.9
ELS20	Chita	7721	556	41	22	1.3	2.5	0.4	0.8	61.9	36.4	41.0	21.2
Mean (Chita samples)		13555.5 ± 3041.0	5258.7 ± 3728.7	53.6 ± 16.9	23.7 ± 11.8	1.1 ± 0.7*	1.3 ± 1.0	0.3 ± 0.2*	0.4 ± 0.3	78.0 ± 17.9*	41.7 ± 24.8	53.6 ± 16.9*	23.6 ± 11.8

‘N.F.’ denotes for ‘not found’. *:*p < 0.05* (by Mann-Whitney test) was considered significantly different between the two sample groups.

### Comparative analysis of bacterial composition in the NFCM of Kalmykia and Chita

**Figure 1:**
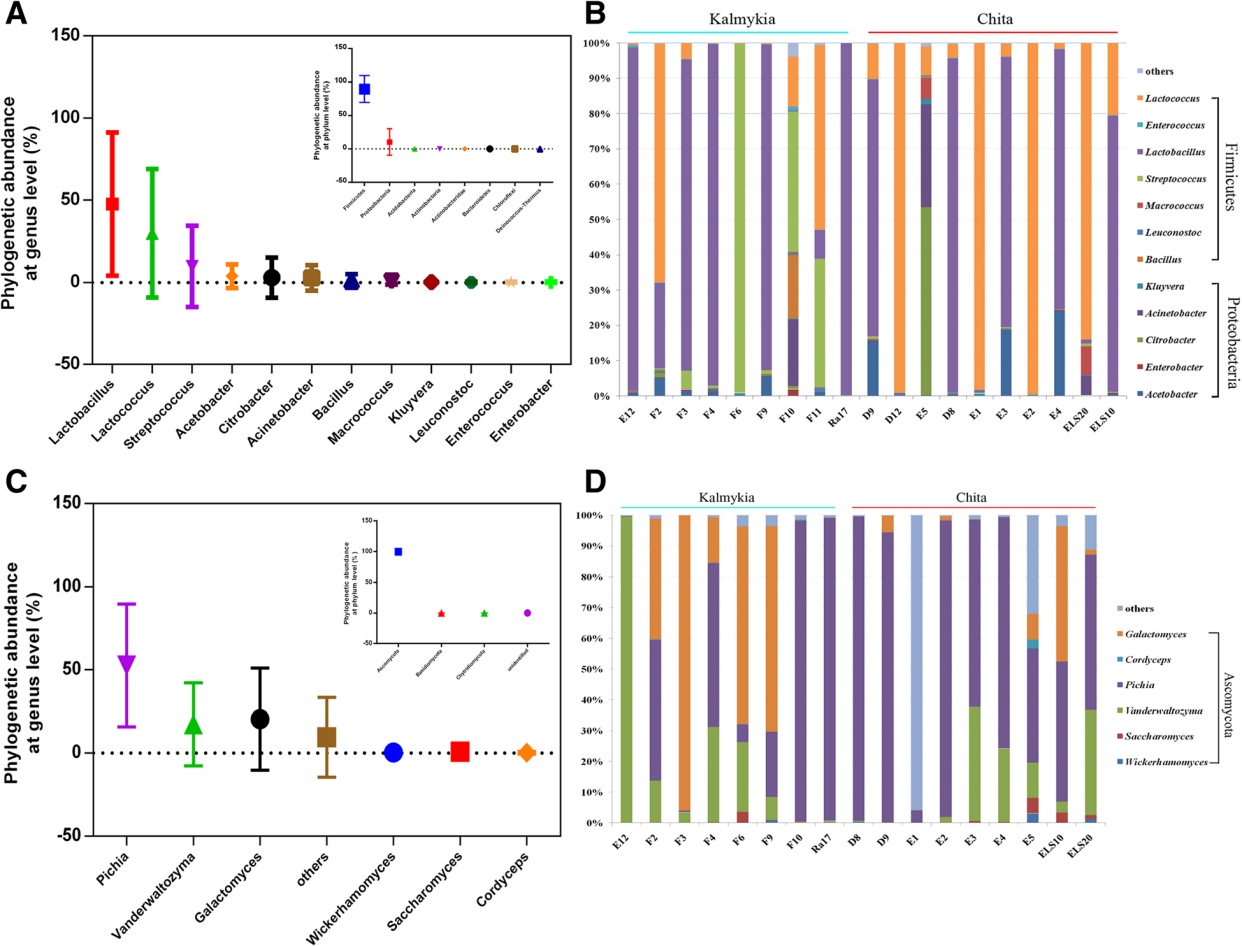
**Relative abundance and diversity of bacteria (A, B) and fungi (C, D) in the samples.**

Seven bacterial phyla were identified in the NFCM sampled in this study (Figure 1A), namely Firmicutes, Proteobacteria, Actinobacteria, Bacteroidetes, Acidobacteria, Chloroflexi and Deinococcus-Thermus. The predominant phylum was Firmicutes, accounting for 89.62% of the total bacterial sequences. This phylum contributed to 95.07% and 84.71% of the bacterial sequence reads of the Kalmykia and Chita samples, respectively. Proteobacteria was the subdominant phylum, which contributed to 10.33% of the total bacterial sequences. Minor phyla, including Actinobacteria, Bacteroidetes, Acidobacteria, Chloroflexi and Deinococcus-Thermus, together contributed to 0.04% of the total bacterial sequences. Only the phylum Acidobacteria, but not the other six phyla, showed a significant difference between the Kalmykia and Chita samples (Table 2).

**Table 2:** **Phylum and genera showing significant difference between the two sample groups**

	**Relative contribution (%)**		**Median, range (%)**		**P-value**
	**Kalmykia**	**Chita**	**Kalmykia**	**Chita**	
**Phylum**					
Acidobacteria	0.009	0.034	0.007, 0-0.042	0.02, 0-0.09	0.02
**Genus**					
*Aeromonas*	0.139	0.000	0.008, 0 -1.197	0	0.022
*Bacillus*	2.036	0.000	0.059, 0-18	0	0.006
*Clostridium*	0.015	0.001	0.016, 0-0.042	0, 0-0.007	0.006
*Streptococcus*	20.284	0.278	3.046, 0.065-99.044	0.241, 0-0.785	0.022
*Vogesella*	0.007	0.000	0.008, 0-0.021	0	0.022
*Lactococcus*	15.520	42.877	2.627, 0.021-67.581	20.418, 1.783-99.552	0.043

The major bacterial genera in NFCM mainly belonged to the phyla, Firmicutes (*Lactobacillus*, *Lactococcus*, *Streptococcus*, *Macrococcus*, *Leuconostoc*, *Enterococcus* of 47.69%, 29.91%, 9.75%, 0.82%, 0.14% and 0.13%, respectively) and Proteobacteria (*Acetobacter*, *Citrobacter*, *Acinetobacter*, *Bacillus*, *Kluyvera*, *Enterobacter* of 3.94%, 2.92%, 2.87%, 0.96%, 0.26%, 0.12%, respectively) (Figure 1A). In addition, the percentages of sequence reads of 12 genera were different between the two sample groups. *Lactobacillus* contributed to more than 70% in most samples, except for F2, F11, ELS20, E1, D12, F10, E5, E2, and F6. *Lactococcus* was dominant in the samples, E2, D12, E1, ELS20, F2, and F11, contributing to more than 50% of total bacterial sequences (Figure 1B). However, considerable difference was noted between the bacterial compositions of the two groups. Six bacterial genera were found to be significantly differing between the Kalmykia (enrichment of *Aeromonas*, *Bacillus*, *Clostridium*, *Streptococcus*, *Vogesella*; p values ranged from 0.006 to 0.022) and Chita (enrichment of *Lactococcus*; p = 0.043) samples (Table 2).

**Figure 2:**
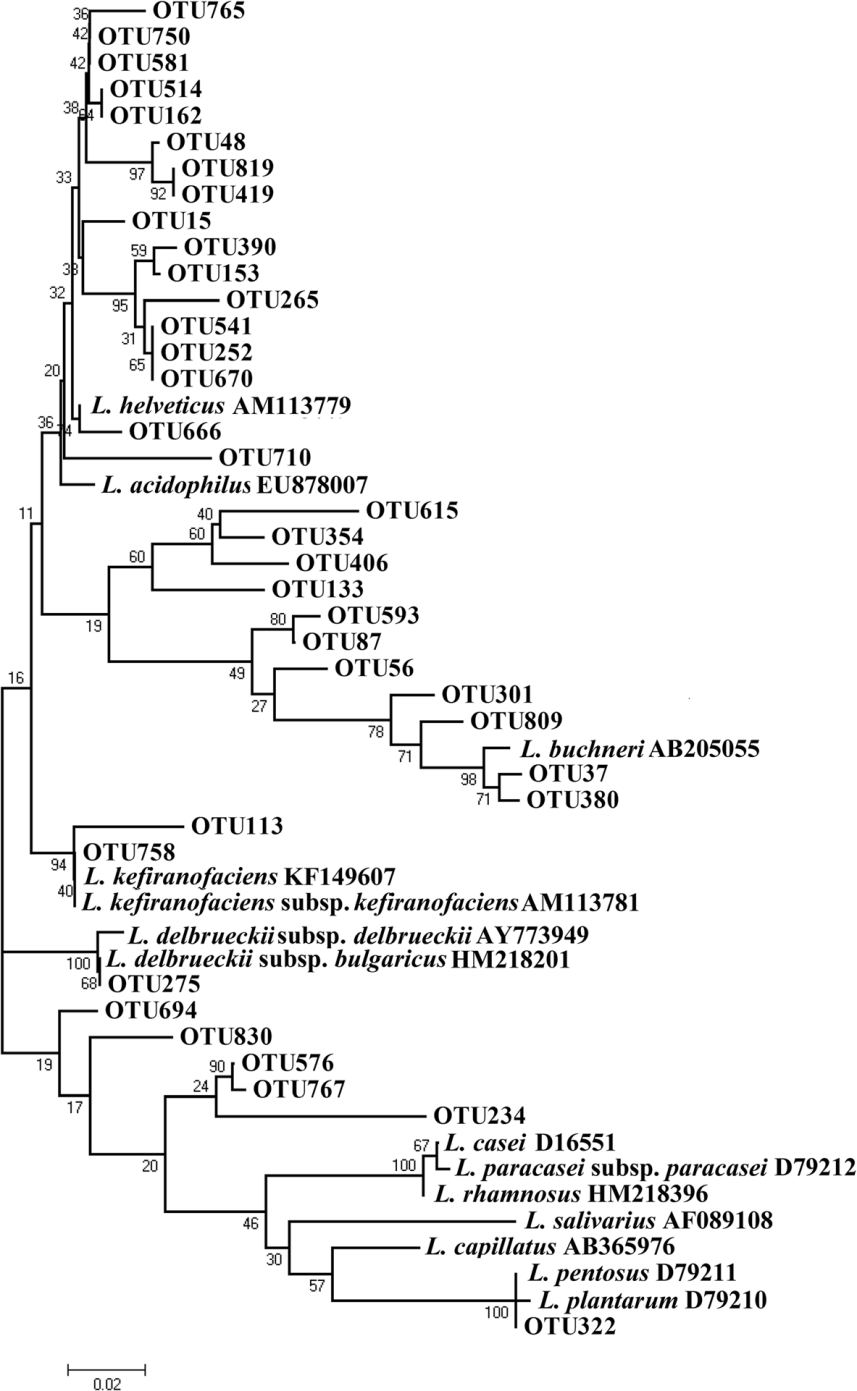
**Phylogenetic tree constructed based on the** ***Lactobacillus*** **OTU sequences.** Reference sequences from the species *L. helveticus*, *L. acidophilus*, *L. buchneri*, *L. kefiranofaciens*, *L. kefiranofaciens* subsp. *kefiranofaciens*, *L. delbrueckii* subsp. *delbrueckii*, *L. casei*, *L. paracasei* subsp. *paracasei*, *L. rhamnosus*, *L. salivarius*, *L. capillatus*, *L. pentosus*, *L. plantarum* were retrieved from the NCBI database.

The most abundant genus, *Lactobacillus*, was comprised of 37 different types of OTUs (Figure 2). Based on the phylogenetic tree, some of these *Lactobacillus* OTUs could be further assigned to different *Lactobacillus* species. For example, OTU666 and OTU275 clustered with *L. helveticus* and *L. delbrueckii* subsp. *bulgaricus* respectively. OTU758 was closest to *L. kefiranofaciens* and *L. kefiranofaciens* subsp. *kefiranofaciens*. OTU322 were nearest to *L. pentosus* and *L. plantarum*. OTU37 and OTU380 affiliated to *L. buchneri*, meaning that they were likely to be the subspecies of *L. buchneri*.

### Multivariate analysis of the bacterial communities of NFCM from Kalmykia and Chita

**Figure 3:**
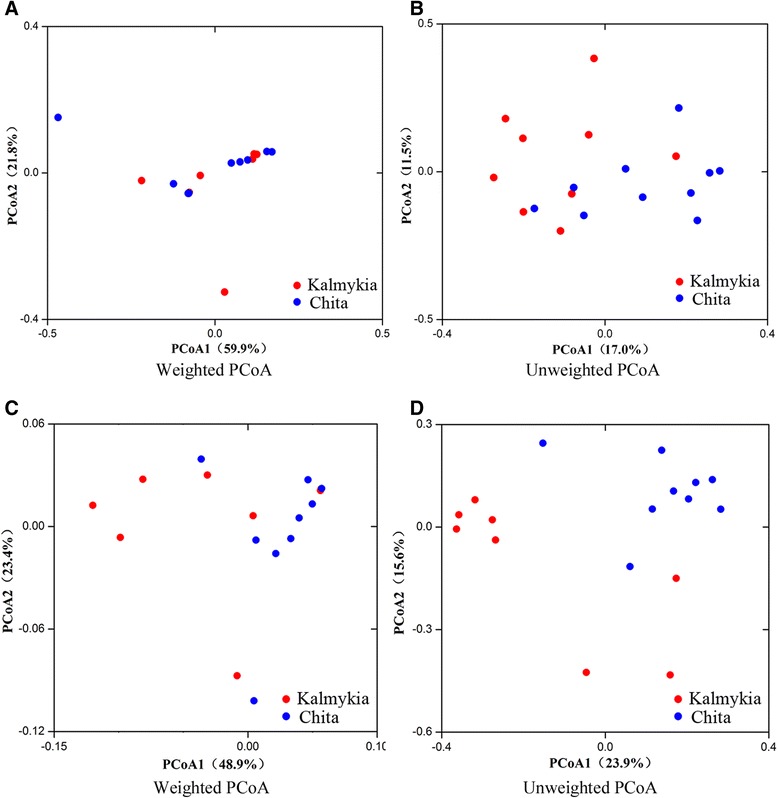
**UniFrac principal coordinate analysis of the bacterial (A, B) and fungal (C, D) communities in the NFCM samples.** Red and blue symbols represent samples from Kalmykia and Chita, respectively.

PCoA and MANOVA were performed to analyze the bacterial communities and structure of the two sample groups (Figure 3A and B). PCoA based on the weighted (accounted for 59.9% and 21.8% of the total variance) (Figure 3A) and the unweighted (accounted for 17.0% and 11.5% of the total variance) (Figure 3B) UniFrac distances revealed the existence of bacterial structural difference, as symbols representing the two groups were largely separated on the unweighted but not weighted PCoA score plot. This result suggests that the difference lied not on the abundant OTUs. Even though there was overlapping of data points from the Kalmykia and Chita samples on both score plots, the results of MANOVA based on both the weighted (p < 0.05) and unweighted (p < 0.05) UniFrac distance confirmed that significant differences existed.

**Figure 4:**
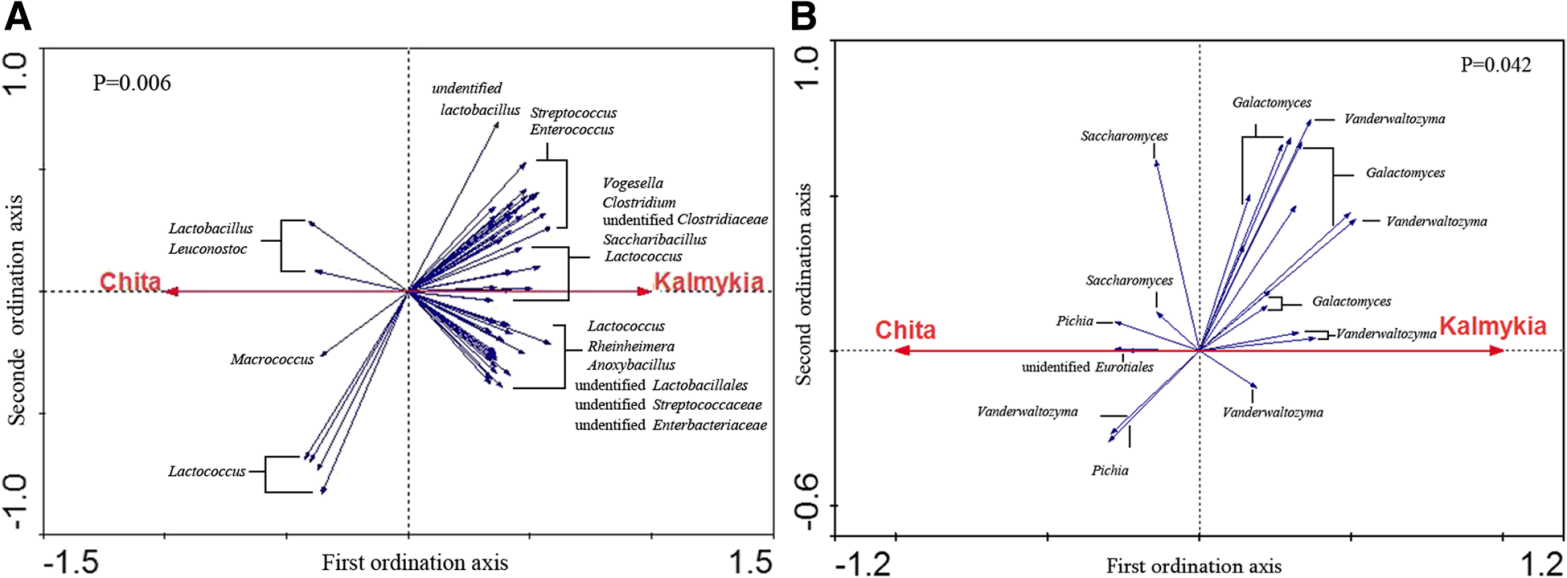
**Biplot of redundancy analysis (RDA) of the bacteria (A) and fungi (B) in the NFCM from Chita and Kalmykia.** Red arrows represent the constrained explanatory variables, Kalmykia and Chita. Blue arrows represent the response variables with the first ordination axis explaining respectively for at least 8.6% and 1.8% of the variability of the bacterial and fungal microbiota. The p-values generated from the Monte Carlo Permutation Test are included in the plots.

To further identify the bacterial populations that accounted for the difference observed between the Kalmykia and Chita NFCM samples, RDA was carried out (Figure 4A). In this case, NFCM samples in Kalmykia and Chita were the “nominal environmental variables”, and the relative abundances of all OTUs (at 97% similarity) were the response variables. Monte Carlo Permutation Test (MCPT) showed that the constrained ordination model was statistically significant (p =0.006) and the first canonical axis was able to explain 8.6% of the variability of response variables (Figure 4A). Moreover, 60 OTUs were identified as the key variables, which had good correlation with sample scores on the RDA canonical axis (Figure 4A). Among them, 7 OTUs (mainly, *Lactobacillus*, *Leuconostoc*, *Macrococcus* and *Lactococcus*) were enriched in the samples from Chita, while the other 53 OTUs (mainly, *Streptococcus*, *Enterococcus*, *Clostridium*, *Saccharibacillus* and *Anoxybacillus*) were enriched in the Kalmykia samples.

### Fungal composition of the NFCM

The major fungal phyla found in the NFCM were Ascomycota, Basidiomycota and Chytridiomycota, constituting 99.77%, 0.008% and 0.002% of the fungal population (Figure 1C), respectively. No fungal sequence read was detected in the samples, F11 and D12, which may due to an intrinsic low abundance of fungi present in these samples. At the genus level, 20 fungal genera were identified, among which six genera were highly variable between samples, namely, *Pichia*, *Galactomyces*, *Vanderwaltozyma*, *Wickerhamomyces*, *Saccharomyces*, and *Cordyceps* (representing 52.66%, 20.45%, 17.38%, 0.37%, 0.93%, and 0.24% of the fungal population) (Figure 1D). Among them, *Pichia*, *Galactomyces* and *Vanderwaltozyma* were the dominant fungal genera (at least 1.0% of the total fungal gene pool) across all samples. *Pichia* contributed to more than 50% in most samples, except for the samples, ELS10, F2, E5, F9, F6, E1, F3, and E12, while *Galactomyces* was prevalent in the samples, F3, F6, and F9, contributing to more than 50% of total fungal sequences. Moreover, in the sample E12, *Vanderwaltozyma* contributed to 99% of total fungal sequences.

### Comparison of the fungal composition between the Kalmykia and Chita samples

The weighted and unweighted UniFrac PCoA was performed to evaluate if any structural difference existed in the fungal community between the two sample groups (Figure 3C and D). Although there was slight overlapping between the samples from the two groups on the score plots, data points were largely separated by both weighted (accounted for 48.9% and 23.4% of the total variance by the first two PCs) (Figure 3C) and unweighted (accounted for 29.3% and 15.6% of the total variance by the first two PCs) (Figure 3D) UniFrac analysis. However, a further MANOVA test showed that only the unweighted (p < 0.05) but not weighted (p > 0.05) UniFrac distance showed significant difference between sample groups. These results suggest that the structural difference in the fungal community was attributed to the less abundant fungal lineages.

RDA was applied to further identify the specific fungal groups that accounted for the difference observed between the Kalmykia and Chita NFCM samples (Figure 4B). MCPT showed that the constrained ordination model by Kalmykia or Chita was significant (P = 0.042). We identified 21 OTUs that fitted well by the sample scores on the canonical axis as key variables; each had at least 1.8% of the variability in its values explained by this axis. On the RDA ordination plot, 6 OTUs were enriched in Chita NFCM samples, and they belonged to the genera *Saccharomyces*, *Pichia*, *Vanderwaltozyma* and unidentified *Trichocomaceae*. Meanwhile, 15 OTUs were enriched in Kalmykia NFCM samples, which mainly belonged to the genera *Vanderwaltozyma* and *Galactomyces*.

## Discussion

Pyrosequencing analysis is able to provide a thorough description of microbiota community in environmental samples, which also helps to reveal their potential function. In the current study, the bacterial and fungal communities of NFCM from Kalmykia and Chita were studied. Firmicutes, Proteobacteria, Bacteroidetes and Actinobacteria were the dominant bacteria at phylum level, while the abundance of Acidobacteria was considerably different between the two groups. Firmicutes was the most abundant phylum, which was in line with results from some previous studies performed in naturally fermented dairy products, such as kefir [10], fermented seafood [19], kochujang [20], pearl millet slurries [23], and artisanal cheeses [17]. The fungal community was dominated with Ascomycota, and this phylum was prevalent in Korean alcoholic beverages [24] and Chinese liquors [25]. As reflected by the Shannon index and rarefaction analysis, the bacterial diversity and richness were higher than that of the fungi in the same environment, which is consistent with a previous report on tarag [1]. The report found that the isolated number and density of lactic acid bacteria were considerably higher than that of yeasts in the studied traditional Mongolian fermented dairy products.

*Lactobacillus* and *Lactococcus* were the dominant genera for samples from both Kalmykia and Chita. These two genera are commonly associated with naturally fermented dairy products [1,11,26]. *Lactobacillus* was the predominant genus in our samples. The starter cultures of the homemade NFCM in this study were passage from the old NFCM. It was possible that the high proportion of *Lactobacillus* was retained and even enriched during NFCM fermentation. It has been reported that the acid tolerance of lactobacilli is higher than *Streptococcus* and *Lactococcus* [27]. Some species of *Lactococcus* have low acid tolerance, however, they could be isolated from raw milk and were found growing during the early stage of fermentation [11,21]. These may explain the lower abundance of *Lactococcus* than *Lactobacillus* as seen in our results.

Some reports claim that 16S rRNA is a slowly diverging molecule and it fails to reveal significant differences between recently diverged species; thus protein-encoding genes (such as recA gene for *L. plantarum* and *L. casei* [28-30]) may be used as the phylogenetic markers to ensure an accurate assignment of OTUs to the species or even subspecies level. Our results demonstrate a high resolution of pyrosequencing of 16S rRNA gene in assigning *Lactobacillus* to the precision of species and subspecies levels. Based on phylogenetic grouping of *Lactobacillus* OTU representative sequences, *L. helveticus* and *L. delbrueckii* subsp. *bulgaricus* were predominant in the NFCM. A similar *Lactobacillus* composition was reported in the traditional dairy products, airag and tarag [1,8].

Furthermore, the major genera (each comprised over 0.1% of total bacterial sequences), *Streptococcus*, *Macrococcus*, *Leuconostoc*, *Enterococcus*, *Acetobacter*, *Citrobacter*, *Acinetobacter*, *Bacillus*, *Kluyvera*, *Enterobacter*, were present in our NFCM samples. Among them, *Streptococcus*, *Macrococcus*, *Leuconostoc*, and *Enterococcus* have been reported as the subdominant genera in raw milk [5] and cheese [4], which aligned with the results of our study. *Acinetobacter* and *Enterobacter* were detected in Mexican alcoholic beverages. It was speculated that these genera were originated from the bacterial contamination in raw milk; and they subsequently decreased during the fermentation process [31].

*Pichia*, *Galactomyces* and *Vanderwaltozyma* were the dominant fungal genera in NFCM. *Pichia* is commonly associated with fermentative metabolic processes of naturally fermented dairy products and cheese [1,32,33], as this genus takes part in hydrolyzing milk protein and fat, assimilating lactic acid, and producing ethanol [34]. *Galactomyces* has been reported as the dominant yeasts in dairy products, which may involve in lactic acid utilization, proteolysis, lipolysis and flavour development [35]. However, a previously published study reported a different makeup of fungal community in two types of traditional Mongolian fermented products namely airag and tarag. Airag was dominated with the species *Kluyveromyces marxianus*, while the prevalent species in tarag were *Saccharomyces cerevisiae*, *Issatchenkia orientalis*, and *Kazachstania unispora* [1].

Our data revealed clear differences in the microbiota of NFCM collected in the two sampling locations. However, at this point, the exact reasons for the observed variation in the microbiota have not been identified. Similar to our study, previous reports have found that the exposure of cheese to different external environments during the manufacturing process impacted the microbiota composition of the final products [17,36]. The microbial communities of traditional fermented products, such as fermented milk [37] and kochujiang [20], varied with the sampling geographic or manufacturing region. Moreover, animals living at different altitudes, e.g. yak in the Tibetan region, may have different resistance to cold environment, leading to an unusual microbiota makeup of yak milk and its fermented products [38-40]. Kalmykia and Chita are located 6,165 kilometers apart in southern Russia. The NFCM collected from both sites were produced with the same manufacturing process. However, the two places have dissimilar altitude and climate characteristics. Kalmykia is located at the East European Plain, with averages of about 170 meters in elevation [41]. It has a continental climate, with very hot and dry summers (average July temperature is +24°C) and cold winters (average January temperature is −5°C) with little snow (average annual precipitation of up to 400 millimeters). On the contrary, Chita is located at the Central Siberian Plateau and the average elevation is about 500-700 meters [41]. It is generally colder in Chita than Kalmykia with the average summer and winter temperatures ranged from +19°C and −16°C, respectively. Additionally, Chita is comparatively humid than Kalmykia with an average annual precipitation of up to 500 millimeters. Thus, we speculate that the factor of geographic environment including altitudes and climate play a more significant role over the manufacturing process in resulting in the different microbiota compositions of the analyzed products. Our unpublished results revealed a higher microbial richness in the Mongolian style fermented products made by similar methods collected in Russia as compared with those from Mongolia and Inner Mongolia of China. This further confirms that the variation in the microbiota structure of these fermented products may be regional-based.

Some other crucial factors that may affect the fermented milk microbiota composition are the hygienic quality of the milk and the manufacturing process, as well as the back slopping technique. These factors may together contribute to the selection of specific microbial groups and affect the ecological succession of the microbial communities during the fermentation process.

## Conclusions

To sum up, the current study analyzed the bacterial and fungal community diversity of NFCM in Kalmykia and Chita. Our results showed that clear structural differences existed in the microbiota of NFCM from Kalmykia and Chita. The current study has provided interesting insights into the relationship between the microbial composition of NFCM and their geographic regions. The results have also shown that pyrosequencing technique is useful for detecting a wide diversity of microorganisms in NFCM. The results obtained here will be valuable for screening for beneficial strains from traditional fermented dairy products, which are made by the Mongolians residing in Russia. Our study has also provided novel data for Mongolian traditional fermented dairy products.

## Methods

### Sample collection

A total of nineteen NFCM samples were collected from Russia, including nine samples (E12, F10, F2, F3, F4, F6, F9, F11, Ra17) from the Republic of Kalmykia and ten samples (D8, D9, D12, E1, E2, E3, E4, E5, ELS10, ELS20) from Chita. The distance between Kalmykia and Chita is about 6,165 kilometers (Additional file 2: Figure S2). All the NFCM was made in a similar way by natural fermentation of cow’s milk in a special traditional container. Firstly, the raw cow’s milk was boiled and cooled, followed by the step of skimming of lipids. An enrichment starter was then inoculated in the milk by back-slopping. The mixture was allowed to ferment overnight at ambient temperature. Samples were collected aseptically and were kept in ice boxes during transportation. The sample sequence information was listed in Table 1.

### DNA extraction and PCR amplification

Genomic DNA was extracted from each sample using Qiagen DNA Stool Mini Kit (Qiagen, Hilden, Germany) following the manufacturer’s instruction. The quality of extracted DNA was checked by 0.8% agarose gel electrophoresis and spectrophotometry (optical density at 260nm/280nm ratio). All extracted DNA samples were stored at -20°C for further analysis.

The V1-V3 region of bacterial 16S rRNA and V4 region of fungal 18S rRNA were amplified by PCR for barcoded pyrosequencing. The 16S rRNA gene V1-V3 region of bacteria was amplified using the universal forward 27F (5’-AGAGTTTGATCCTGGCTCAG-3’) and the reverse 533R (5’- TTACCGCGGCTGCTGGCAC-3’) primers [42]. The 18S rRNA gene V4 region of fungi targeted at the domain fungi was amplified with the forward 3NDF (5’-GGCAAGTCTGGTGCCAG-3’) and the reverse V4_euk_R2 (5’-ACGGTATCT(AG)ATC(AG)TCTTCG-3’) primers [43]. These primers contained a set of 6-nucleotide barcodes. PCR amplification of the 16S rRNA V1-V3 and 18S rRNA V4 regions were performed, as described previously [23]. The PCR program was as follows: 95°C for 2 min; 30 cycles of 95°C for 30 s, 55°C for 30 s, and 72°C for 30 s with a final extension of 72°C for 5min. The amplicon mixture was applied to the Genome Sequencer FLX454 Titanium System (454 Life Sciences). The quality control for PCR amplification, sequence preprocessing and raw datum processing were performed, as described previously [17].

### Pyrosequencing and bioinformatics processing

The extraction of high-quality sequences was firstly performed with the QIIME package (Quantitative Insights Into Microbial Ecology) (v1.2.1). Raw sequences were selected based on sequence length, quality, primer and tag. According to the following criteria [44,45], low-quality sequences were removed: (i) raw reads were shorter than 110 nucleotides, (ii) sequences had imperfect matches to the barcode, (iii) sequences displayed a fuzzy match to at least one end of primers, (iv) sequences with only a short variable region of less than 100 nucleotides, (v) raw reads that had a quality score of <20 in more than 7% of the bases in the variable region. PyNAST [46] and UCLUST [47] were then applied to align the extracted high-quality sequences under 100% clustering of sequence identity in order to obtain representative sequences. The unique sequence set was classified into operational taxonomic units (OTUs) under the threshold of 97% identity using UCLUST after the selection of the representative sequences. ChimeraSlayer was applied to remove the potential chimeric sequences in the representative set of OTUs [48]. The taxonomy of each OTU representative sequence was performed using the Ribosomal Database Project (RDP) II database [49] that classified at a minimum bootstrap threshold of 80%. OTUs occurred only once or twice were discarded. A *de novo* taxonomic tree was constructed employing a representative chimera-checked OTU set in FastTree for downstream analysis [50], including the beta diversity calculation. The Shannon-Wiener, Simpson’s diversity, the Chao1 and rarefaction estimators were calculated in order to evaluate the alpha diversity. UniFrac distance [51] was based on the phylogenetic tree. Both weighted and unweighted calculations were performed for the principal coordinate analysis (PCoA).

### Statistical analyses

Statistical analyses were performed mainly under Matlab® environment (The MathWorks, Natick, MA, USA) and using the software Canoco for Windows 4.5 (Microcomputer Power, NY, USA). Rarefaction analysis, Shannon diversity index and Simpson’s diversity index were used to estimate the richness and diversity of OTUs. Principal coordinate analysis (PCoA) was used to assess the microbiota structure of different samples. Redundancy analysis (RDA) was applied to identify microbial groups that significantly contributed to the structural difference. Differences in the relative abundances of taxonomic groups at phylum and genus levels between samples were evaluated with Mann–Whitney test. P-values of less than 0.05 were considered significantly different between sample pairs. The phylogenetic grouping of *Lactobacillus* representative sequences was performed according to Felis and Dellaglio [52] and was constructed using MAGE 5.0 [53].

### Nucleotide sequence accession numbers

The sequence data reported in this study have been deposited in the MG-RAST database (accession No. 4570605.3–4570640.3).

## Additional files

Additional file 1: Figure S1. Rarefaction analysis and Shannon diversity estimates of the pyrosequencing reads of bacteria (A, B) and fungi (C, D) in NFCM. Red and blue lines represent samples from Kalmykia and Chita, respectively. Additional file 2: Figure S2. NFCM sampling sites. Sampling sites are mapped using Esri® ArcMap™ 10.1. The sampling sites of Kalmykia and Chita are shown in green and red, respectively. The distance between the two places is 6,165 km.

## References

[1] Watanabe K, Fujimoto J, Sasamoto M, Dugersuren J, Tumursuh T, Demberel S (2008). Diversity of lactic acid bacteria and yeasts in Airag and Tarag, traditional fermented milk products of Mongolia. World J Microbiol Biotechnol.

[2] Rodrigues KL, Caputo LRG, Carvalho JCT, Evangelista J, Schneedorf JM (2005). Antimicrobial and healing activity of kefir and kefiran extract. Int J Antimicrob Agents.

[3] Golowczyc MA, Gugliada MJ, Hollmann A, Delfederico L, Garrote GL, Abraham AG (2008). Characterization of homofermentative lactobacilli isolated from kefir grains: potential use as probiotic. J Dairy Res.

[4] Casalta E, Sorba J-M, Aigle M, Ogier J-C (2009). Diversity and dynamics of the microbial community during the manufacture of Calenzana, an artisanal Corsican cheese. Int J Food Microbiol.

[5] Delbès C, Ali-Mandjee L, Montel M-C (2007). Monitoring bacterial communities in raw milk and cheese by culture-dependent and-independent 16S rRNA gene-based analyses. Appl Environ Microbiol.

[6] Dobson A, O'Sullivan O, Cotter PD, Ross P, Hill C (2011). High-throughput sequence-based analysis of the bacterial composition of kefir and an associated kefir grain. Fems Microbiol Lett.

[7] Van Hoorde K, Verstraete T, Vandamme P, Huys G (2008). Diversity of lactic acid bacteria in two Flemish artisan raw milk Gouda-type cheeses. Food Microbiol.

[8] Liu W, Bao Q, Qing M, Chen X, Sun T, Li M (2012). Isolation and identification of lactic acid bacteria from Tarag in Eastern Inner Mongolia of China by 16S rRNA sequences and DGGE analysis. Microbiol Res.

[9] Liu W, Sun Z, Zhang Y, Zhang C, Yang M, Sun T (2012). A survey of the bacterial composition of kurut from Tibet using a culture-independent approach. J Dairy Sci.

[10] Chen H-C, Wang S-Y, Chen M-J (2008). Microbiological study of lactic acid bacteria in kefir grains by culture-dependent and culture-independent methods. Food Microbiol.

[11] Alegría Á, Szczesny P, Mayo B, Bardowski J, Kowalczyk M (2012). Biodiversity in Oscypek, a traditional Polish cheese, determined by culture-dependent and-independent approaches. Appl Environ Microbiol.

[12] Abriouel H, Martín-Platero A, Maqueda M, Valdivia E, Martínez-Bueno M (2008). Biodiversity of the microbial community in a Spanish farmhouse cheese as revealed by culture-dependent and culture-independent methods. Int J Food Microbiol.

[13] Friedrich U, Lenke J (2006). Improved enumeration of lactic acid bacteria in mesophilic dairy starter cultures by using multiplex quantitative real-time PCR and flow cytometry-fluorescence in situ hybridization. Appl Environ Microbiol.

[14] Jany J-L, Barbier G (2008). Culture-independent methods for identifying microbial communities in cheese. Food Microbiol.

[15] Margulies M, Egholm M, Altman WE, Attiya S, Bader JS, Bemben LA (2005). Genome sequencing in microfabricated high-density picolitre reactors. Nature.

[16] Masoud W, Takamiya M, Vogensen FK, Lillevang S, Al-Soud WA, Sørensen SJ (2011). Characterization of bacterial populations in Danish raw milk cheeses made with different starter cultures by denaturating gradient gel electrophoresis and pyrosequencing. Int Dairy J.

[17] Quigley L, O'Sullivan O, Beresford TP, Ross RP, Fitzgerald GF, Cotter PD (2012). High-throughput sequencing for detection of subpopulations of bacteria not previously associated with artisanal cheeses. Appl Environ Microbiol.

[18] Leite A, Mayo B, Rachid C, Peixoto R, Silva J, Paschoalin V (2012). Assessment of the microbial diversity of Brazilian kefir grains by PCR-DGGE and pyrosequencing analysis. Food Microbiol.

[19] Roh SW, Kim K-H, Nam Y-D, Chang H-W, Park E-J, Bae J-W (2010). Investigation of archaeal and bacterial diversity in fermented seafood using barcoded pyrosequencing. ISME J.

[20] Nam YD, Park SL, Lim SI (2012). Microbial composition of the Korean traditional food “kochujang” analyzed by a massive sequencing technique. J Food Sci.

[21] An Y, Adachi Y, Ogawa Y (2004). Classification of lactic acid bacteria isolated from chigee and mare milk collected in Inner Mongolia. Anim Sci J.

[22] Uchida K, Hirata M, Motoshima H, Urashima T, Arai I (2007). Microbiota of ‘airag’, ‘tarag’and other kinds of fermented dairy products from nomad in Mongolia. Anim Sci J.

[23] Humblot C, Guyot J-P (2009). Pyrosequencing of tagged 16S rRNA gene amplicons for rapid deciphering of the microbiomes of fermented foods such as pearl millet slurries. Appl Environ Microbiol.

[24] Jung M-J, Nam Y-D, Roh SW, Bae J-W (2012). Unexpected convergence of fungal and bacterial communities during fermentation of traditional Korean alcoholic beverages inoculated with various natural starters. Food Microbiol.

[25] Li X-R, Ma E-B, Yan L-Z, Meng H, Du X-W, Zhang S-W (2011). Bacterial and fungal diversity in the traditional Chinese liquor fermentation process. Int J Food Microbiol.

[26] Bao Q, Yu J, Liu W, Qing M, Wang W, Chen X (2012). Predominant lactic acid bacteria in traditional fermented yak milk products in the Sichuan Province of China. Dairy Sci Technol.

[27] Rogosa M, Mitchell JA, Wiseman RF (1951). A selective medium for the isolation and enumeration of oral and fecal lactobacilli. J Bacteriol.

[28] Felis GE, Dellaglio F, Mizzi L, Torriani S (2001). Comparative sequence analysis of a recA gene fragment brings new evidence for a change in the taxonomy of the Lactobacillus casei group. Int J Syst Evol Microbiol.

[29] Torriani S, Felis GE, Dellaglio F (2001). Differentiation of Lactobacillus plantarum, L. pentosus, and L. paraplantarum by recA gene sequence analysis and multiplex PCR assay with recA gene-derived primers. Appl Environ Microbiol.

[30] Bringel F, Castioni A, Olukoya DK, Felis GE, Torriani S, Dellaglio F (2005). Lactobacillus plantarum subsp. argentoratensis subsp. nov., isolated from vegetable matrices. Int J Syst Evol Microbiol.

[31] Escalante A, Giles-Gómez M, Hernández G, Córdova-Aguilar MS, López-Munguía A, Gosset G (2008). Analysis of bacterial community during the fermentation of pulque, a traditional Mexican alcoholic beverage, using a polyphasic approach. Int J Food Microbiol.

[32] Miyamoto M, Seto Y, Nakajima H, Burenjargal S, Gombojav A, Demberel S (2010). Denaturing gradient gel electrophoresis analysis of lactic acid bacteria and yeasts in traditional Mongolian fermented milk. Food Sci Technol Res.

[33] Rahman N, Xiaohong C, Meiqin F, Mingsheng D (2009). Characterization of the dominant microflora in naturally fermented camel milk shubat. World J Microbiol Biotechnol.

[34] Arakawa K, Miyamoto M, Miyamoto T (2013). Interaction between lactic acid bacteria and yeasts in airag, an alcoholic fermented milk. Anim Sci J.

[35] Wyder M-T, Bachmann H-P, Puhan Z (1999). Role of selected yeasts in cheese ripening: an evaluation in foil wrapped Raclette cheese. LWT-Food Sci Technol.

[36] Feurer C, Vallaeys T, Corrieu G, Irlinger F (2004). Does smearing inoculum reflect the bacterial composition of the smear at the end of the ripening of a French soft, red-smear cheese?. J Dairy Sci.

[37] Baldorj R, Tumenjargal D, Batjargal B (2003). Biochemical and Microbiological Study of Fermented Mare’s Milk (Airag) Prepared by TRADITIONAL MONGOLIAN TECHNOLOGY. Proceedings of International Scientific Symposium on Nomadic Cultural Tradition: Mongolian Dairy Products.

[38] Ding L, Long R, Shang Z, Wang C, Yang Y, Xu S (2008). Feeding behaviour of yaks on spring, transitional, summer and winter pasture in the alpine region of the Qinghai–Tibetan plateau. Appl Anim Behav Sci.

[39] Wu X-H, Luo Z, Yu L, Ren F-Z, Han B-Z, Nout MR (2009). A survey on composition and microbiota of fresh and fermented yak milk at different Tibetan altitudes. Dairy Sci Technol.

[40] Hu S, Wei H, Guo S, Li L, Hou Y (2011). Flavor evaluation of yak butter in Tsinghai‐Tibet Plateau and isolation of microorganisms contributing flavor. Anim Sci J.

[41] Britannica E (2009). Encyclopædia britannica.

[42] Huse SM, Dethlefsen L, Huber JA, Welch DM, Relman DA, Sogin ML (2008). Exploring microbial diversity and taxonomy using SSU rRNA hypervariable tag sequencing. PLoS Genet.

[43] Bråte J, Logares R, Berney C, Ree DK, Klaveness D, Jakobsen KS (2010). Freshwater Perkinsea and marine-freshwater colonizations revealed by pyrosequencing and phylogeny of environmental rDNA. ISME J.

[44] Zhang J, Wang L, Guo Z, Sun Z, Gesudu Q, Kwok L (2014). 454 pyrosequencing reveals changes in the faecal microbiota of adults consuming Lactobacillus casei Zhang. FEMS Microbiol Ecol.

[45] Zhang J, Guo Z, Lim AAQ, Zheng Y, Koh EY, Ho D (2014). Mongolians core gut microbiota and its correlation with seasonal dietary changes. Sci Rep.

[46] Caporaso JG, Bittinger K, Bushman FD, DeSantis TZ, Andersen GL, Knight R (2010). PyNAST: a flexible tool for aligning sequences to a template alignment. Bioinformatics.

[47] Edgar RC (2010). Search and clustering orders of magnitude faster than BLAST. Bioinformatics.

[48] Haas BJ, Gevers D, Earl AM, Feldgarden M, Ward DV, Giannoukos G (2011). Chimeric 16S rRNA sequence formation and detection in Sanger and 454-pyrosequenced PCR amplicons. Genome Res.

[49] Cole JR, Chai B, Farris RJ, Wang Q, Kulam-Syed-Mohideen A, McGarrell D (2007). The ribosomal database project (RDP-II): introducing myRDP space and quality controlled public data. Nucleic Acids Res.

[50] Price MN, Dehal PS, Arkin AP (2009). FastTree: computing large minimum evolution trees with profiles instead of a distance matrix. Mol Biol Evol.

[51] Lozupone C, Knight R (2005). UniFrac: a new phylogenetic method for comparing microbial communities. Appl Environ Microbiol.

[52] Felis GE, Dellaglio F (2007). Taxonomy of lactobacilli and bifidobacteria. Curr Issues Intest Microbiol.

[53] Tamura K, Peterson D, Peterson N, Stecher G, Nei M, Kumar S (2011). MEGA5: molecular evolutionary genetics analysis using maximum likelihood, evolutionary distance, and maximum parsimony methods. Mol Biol Evol.

