# Supplementary figures and images for: High-throughput sequencing for the detection of the bacterial and fungal diversity in Mongolian naturally fermented cow’s milk in Russia
Source: BMC Microbiol. 2015 Feb 22;15:45. doi: 10.1186/s12866-015-0385-9 (PMC4345014; doi:10.1186/s12866-015-0385-9)

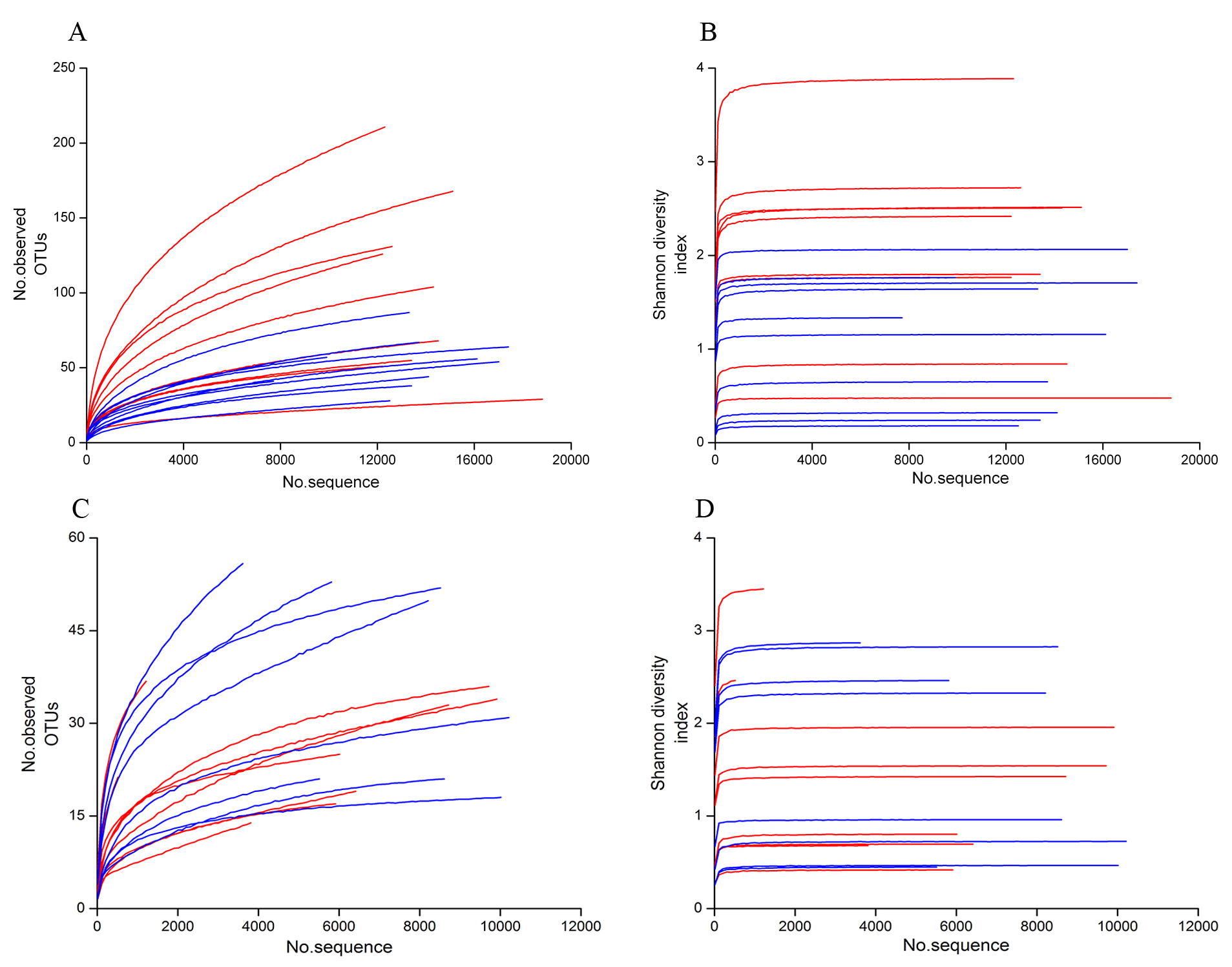

Supplement: Additional file 1: Figure S1. — Rarefaction analysis and Shannon diversity estimates of the pyrosequencing reads of bacteria (A, B) and fungi (C, D) in NFCM. Red and blue lines represent samples from Kalmykia and Chita, respectively. [file 12866_2015_385_MOESM1_ESM.tiff]

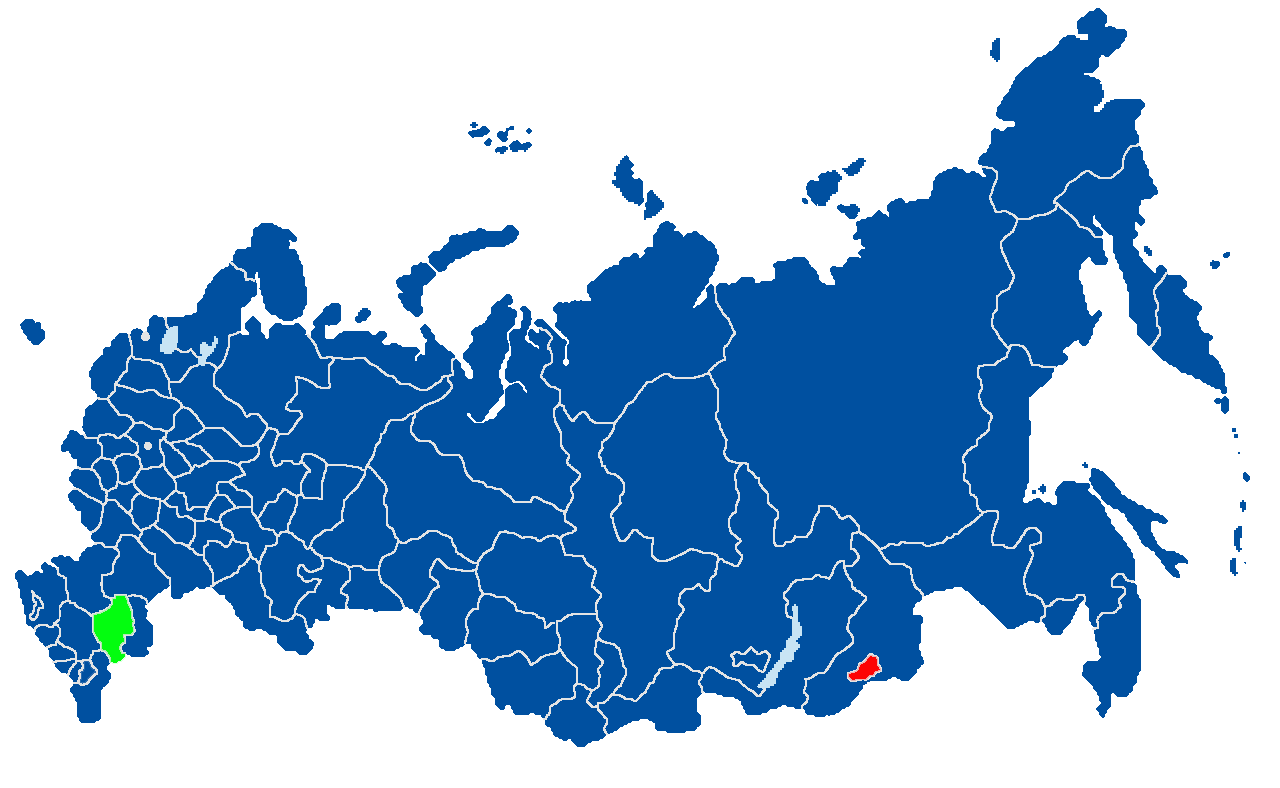

Supplement: Additional file 2: Figure S2. — NFCM sampling sites. Sampling sites are mapped using Esri® ArcMap™ 10.1. The sampling sites of Kalmykia and Chita are shown in green and red, respectively. The distance between the two places is 6,165 km. [file 12866_2015_385_MOESM2_ESM.png]
